# Supplementary material for: Large socioeconomic gap in period life expectancy and life years spent with complications of diabetes in the Scottish population with type 1 diabetes, 2013–2018
Source: PLoS One. 2022 Aug 11;17(8):e0271110. doi: 10.1371/journal.pone.0271110 (PMC9371295; doi:10.1371/journal.pone.0271110)
Supplement: S1 Table — (DOCX) [file pone.0271110.s001.docx]

**S1 Table: Definition of all diabetes-related complications examined in the study.**

**Note:** Information on cardiovascular disease are based on ICD-9/ICD-10 hospital admission codes in the SMR01 register. The definitions of retinopathy/maculopathy, chronic kidney disease, and diabetic foot are based on screening results carried out in predominantly primary care. For the latter three conditions, no further ICD codes from the SMR01 register were used.

| Complication | Definition | ICD9.ICD10.Codes. |
| --- | --- | --- |
| Retinopathy/Maculopathy | Ever diabetic eye screening resulting in | (not applicable) |
|  | referral for maculopathy (M2) or retinopathy (R3/R4) |  |
| Chronic Kidney Disease | Ever eGFR < 60 mL/min/1.73 m2 or | (not applicable) |
|  | renal replacement therapy |  |
| Diabetic Foot | Ever high foot-risk score or  ever active ulcers or amputations | (not applicable) |
| Cardiovascular Disease | Ever hospital admission for: |  |
|  | ischemic heart disease | 411-414 / I20-I25 |
|  | peripheral arterial disease | 443.9, 250.7 / I73.9, E10.5, E11.5, E12.5, E13.5, E14.5 |
|  | transient ischemic attack | 435 / G45 |
|  | myocardial infarction, | 410, 412 / G45, |
|  | cardiac arrhythmia | 427 / I48-I49, |
|  | heart failure | 428 / I11.0, I13.0, I13.1, I13.2, I50 |
|  | hypertension | 401-405 / I10-I15, |
|  | cerebrovascular disease | 430-434 / I60-I69 |
