# Supplementary material for: Large socioeconomic gap in period life expectancy and life years spent with complications of diabetes in the Scottish population with type 1 diabetes, 2013–2018
Source: PLoS One. 2022 Aug 11;17(8):e0271110. doi: 10.1371/journal.pone.0271110 (PMC9371295; doi:10.1371/journal.pone.0271110)
Supplement: S2 Table — (DOCX) [file pone.0271110.s002.docx]

**S2 Table: Fraction of missing information for the study population (in percent) at study entry on 01 January 2013, before the usage of multiple imputation methods.**

| Variable | Percent |
| --- | --- |
| Serialno | 0.00 |
| Slice | 0.00 |
| Age | 0.00 |
| Diabetes Duration | 0.00 |
| Sex | 0.00 |
| SIMD 2016 | 11.33 |
| HbA1c | 0.09 |
| HDL | 1.77 |
| LDL | 23.31 |
| Tchol | 0.11 |
| SBP | 0.03 |
| DBP | 0.03 |
| BMI | 0.22 |
| eGFR | 0.40 |
| Referable Eye Disease Grading | 6.62 |
| Footrisk Score | 1.90 |
| Smoking Status | 0.56 |
| Previous Admission for Cardiovascular Disease | 0.00 |
| Previous Admission for Hypoglycemia | 0.00 |
| Previous Admission for DKA | 0.00 |
