# Supplementary material for: Large socioeconomic gap in period life expectancy and life years spent with complications of diabetes in the Scottish population with type 1 diabetes, 2013–2018
Source: PLoS One. 2022 Aug 11;17(8):e0271110. doi: 10.1371/journal.pone.0271110 (PMC9371295; doi:10.1371/journal.pone.0271110)
Supplement: S3 Table — (DOCX) [file pone.0271110.s003.docx]

**S3 Table: Overview of all utilized transition-specific models used for the main analysis. Models of set 1 were used to derive estimates for all males and all females while models of set 2 were used to derive estimates for males and females by SIMD quintile.**

| Set | Transition | Distribution | Covariates | Observed Events |
| --- | --- | --- | --- | --- |
| 1 | 1: No Complication 🡪 1 complication | Gompertz | sex | 1146 |
| 1 | 2: No Complication 🡪 Death | Gompertz | sex | 107 |
| 1 | 3: 1 Complication 🡪 2 Complications | Gompertz | sex | 1351 |
| 1 | 4: 1 Complication 🡪 Death | Gompertz | sex | 268 |
| 1 | 5: 2 Complications 🡪 3+ Complications | Gompertz | sex | 930 |
| 1 | 6: 2 Complications 🡪 Death | Gompertz | sex | 566 |
| 1 | 7: 3+ Complications 🡪 Death | Gompertz | sex | 554 |
| 2 | 1: No Complication 🡪 1 complication | Gompertz | sex+simd | 1146 |
| 2 | 2: No Complication 🡪 Death | Gompertz | sex+simd | 107 |
| 2 | 3: 1 Complication 🡪 2 Complications | Gompertz | sex+simd | 1351 |
| 2 | 4: 1 Complication 🡪 Death | Gompertz | sex+simd | 268 |
| 2 | 5: 2 Complications 🡪 3+ Complications | Gompertz | sex+simd | 930 |
| 2 | 6: 2 Complications 🡪 Death | Gompertz | sex+simd | 566 |
| 2 | 7: 3+ Complications 🡪 Death | Gompertz | sex+simd | 554 |
