# Supplementary material for: Large socioeconomic gap in period life expectancy and life years spent with complications of diabetes in the Scottish population with type 1 diabetes, 2013–2018
Source: PLoS One. 2022 Aug 11;17(8):e0271110. doi: 10.1371/journal.pone.0271110 (PMC9371295; doi:10.1371/journal.pone.0271110)
Supplement: S4 Table — (DOCX) [file pone.0271110.s004.docx]

**S4 Table: Distribution of weights (in percent) at ages 45-54 by sex and SIMD quintile on 01 January 2013.**

**Note:** These weights w~j can be interpreted as the prevalence of individuals with no, 1, 2 and 3+ complications at an average age of 50 on 01 January 2013.

| Sex | SIMD | No Complication | 1 Complication | 2 Complications | 3+ Complications |
| --- | --- | --- | --- | --- | --- |
| Females | All Quintiles | 58.17 | 26.81 | 10.16 | 4.81 |
| Males | All Quintiles | 59.49 | 24.78 | 10.65 | 5.05 |
| Females | SIMD 1 | 49.32 | 30.23 | 13.18 | 7.05 |
| Females | SIMD 2 | 52.22 | 29.44 | 12.10 | 6.25 |
| Females | SIMD 3 | 60.83 | 23.41 | 11.38 | 4.38 |
| Females | SIMD 4 | 62.86 | 26.70 | 7.52 | 2.91 |
| Females | SIMD 5 | 67.42 | 23.81 | 5.76 | 3.01 |
| Males | SIMD 1 | 52.88 | 26.21 | 14.24 | 6.67 |
| Males | SIMD 2 | 56.05 | 26.59 | 11.31 | 6.05 |
| Males | SIMD 3 | 58.46 | 24.02 | 12.69 | 4.68 |
| Males | SIMD 4 | 62.43 | 23.72 | 8.35 | 5.50 |
| Males | SIMD 5 | 69.34 | 23.00 | 5.57 | 2.09 |
