# Supplementary material for: Large socioeconomic gap in period life expectancy and life years spent with complications of diabetes in the Scottish population with type 1 diabetes, 2013–2018
Source: PLoS One. 2022 Aug 11;17(8):e0271110. doi: 10.1371/journal.pone.0271110 (PMC9371295; doi:10.1371/journal.pone.0271110)
Supplement: S5 Table — Models of set 1 were used to derive estimates for all males and all females. (DOCX) [file pone.0271110.s005.docx]

**S5 Table: Overview of all utilized transition-specific models of set 1. Models of set 1 were used to derive estimates for all males and all females.**

**Note:** Transitions align directly to Fig 1 - Panel (B).

| Transition | Parameter | est | L95 | H95 | HR | HR.L95 | HR.H95 |
| --- | --- | --- | --- | --- | --- | --- | --- |
| 1 | Shape | 0.04 | 0.03 | 0.05 | NA | NA | NA |
| 1 | Rate | 0.04 | 0.04 | 0.05 | NA | NA | NA |
| 1 | Males (Ref: Females) | 0.18 | 0.06 | 0.30 | 1.19 | 1.06 | 1.35 |
| 2 | Shape | 0.07 | 0.04 | 0.09 | NA | NA | NA |
| 2 | Rate | <0.01 | 0.00 | <0.01 | NA | NA | NA |
| 2 | Males (Ref: Females) | 0.47 | 0.05 | 0.88 | 1.60 | 1.06 | 2.41 |
| 3 | Shape | 0.03 | 0.02 | 0.03 | NA | NA | NA |
| 3 | Rate | 0.06 | 0.05 | 0.07 | NA | NA | NA |
| 3 | Males (Ref: Females) | 0.14 | 0.03 | 0.24 | 1.15 | 1.03 | 1.28 |
| 4 | Shape | 0.07 | 0.06 | 0.08 | NA | NA | NA |
| 4 | Rate | <0.01 | 0.00 | 0.01 | NA | NA | NA |
| 4 | Males (Ref: Females) | 0.50 | 0.25 | 0.75 | 1.65 | 1.29 | 2.12 |
| 5 | Shape | -0.01 | -0.02 | -0.00 | NA | NA | NA |
| 5 | Rate | 0.10 | 0.08 | 0.11 | NA | NA | NA |
| 5 | Males (Ref: Females) | 0.18 | 0.05 | 0.31 | 1.20 | 1.05 | 1.36 |
| 6 | Shape | 0.07 | 0.06 | 0.07 | NA | NA | NA |
| 6 | Rate | 0.01 | 0.01 | 0.02 | NA | NA | NA |
| 6 | Males (Ref: Females) | 0.18 | 0.02 | 0.35 | 1.20 | 1.02 | 1.42 |
| 7 | Shape | 0.05 | 0.04 | 0.06 | NA | NA | NA |
| 7 | Rate | 0.04 | 0.03 | 0.05 | NA | NA | NA |
| 7 | Males (Ref: Females) | 0.14 | -0.02 | 0.31 | 1.16 | 0.98 | 1.37 |
