# Supplementary material for: Large socioeconomic gap in period life expectancy and life years spent with complications of diabetes in the Scottish population with type 1 diabetes, 2013–2018
Source: PLoS One. 2022 Aug 11;17(8):e0271110. doi: 10.1371/journal.pone.0271110 (PMC9371295; doi:10.1371/journal.pone.0271110)
Supplement: S6 Table — (DOCX) [file pone.0271110.s006.docx]

**S6 Table: Overview of all utilized transition-specific models of set 2. Note:** Models of set 2 were used to derive estimates for males and females by SIMD quintile. Transitions align directly to Fig 1 - Panel (B).

| Transition | Parameter | est | L95 | H95 | HR | HR.L95 | HR.H95 |
| --- | --- | --- | --- | --- | --- | --- | --- |
| 1 | Shape | 0.04 | 0.04 | 0.05 | NA | NA | NA |
| 1 | Rate | 0.06 | 0.05 | 0.07 | NA | NA | NA |
| 1 | Males (Ref: Females) | 0.18 | 0.06 | 0.30 | 1.20 | 1.06 | 1.35 |
| 1 | SIMD Q2 (Ref: SIMD Q1) | -0.19 | -0.37 | 0.00 | 0.83 | 0.69 | 1.00 |
| 1 | SIMD Q3 | -0.32 | -0.51 | -0.13 | 0.73 | 0.60 | 0.87 |
| 1 | SIMD Q4 | -0.37 | -0.56 | -0.18 | 0.69 | 0.57 | 0.84 |
| 1 | SIMD Q5 (Least Depr.) | -0.47 | -0.65 | -0.28 | 0.63 | 0.52 | 0.75 |
| 2 | Shape | 0.07 | 0.04 | 0.10 | NA | NA | NA |
| 2 | Rate | <0.00 | 0.00 | 0.01 | NA | NA | NA |
| 2 | Males (Ref: Females) | 0.48 | 0.07 | 0.90 | 1.62 | 1.07 | 2.45 |
| 2 | SIMD Q2 (Ref: SIMD Q1) | -0.60 | -1.14 | -0.06 | 0.55 | 0.32 | 0.94 |
| 2 | SIMD Q3 | -0.71 | -1.24 | -0.17 | 0.49 | 0.29 | 0.84 |
| 2 | SIMD Q4 | -1.48 | -2.17 | -0.79 | 0.23 | 0.11 | 0.45 |
| 2 | SIMD Q5 (Least Depr.) | -1.25 | -1.83 | -0.66 | 0.29 | 0.16 | 0.52 |
| 3 | Shape | 0.03 | 0.02 | 0.04 | NA | NA | NA |
| 3 | Rate | 0.07 | 0.06 | 0.08 | NA | NA | NA |
| 3 | Males (Ref: Females) | 0.14 | 0.03 | 0.24 | 1.15 | 1.03 | 1.28 |
| 3 | SIMD Q2 (Ref: SIMD Q1) | -0.06 | -0.22 | 0.11 | 0.95 | 0.80 | 1.11 |
| 3 | SIMD Q3 | -0.17 | -0.33 | -0.00 | 0.84 | 0.72 | 1.00 |
| 3 | SIMD Q4 | -0.23 | -0.40 | -0.06 | 0.79 | 0.67 | 0.94 |
| 3 | SIMD Q5 (Least Depr.) | -0.40 | -0.57 | -0.23 | 0.67 | 0.56 | 0.80 |
| 4 | Shape | 0.07 | 0.06 | 0.08 | NA | NA | NA |
| 4 | Rate | 0.01 | 0.00 | 0.01 | NA | NA | NA |
| 4 | Males (Ref: Females) | 0.50 | 0.25 | 0.75 | 1.65 | 1.29 | 2.12 |
| 4 | SIMD Q2 (Ref: SIMD Q1) | -0.09 | -0.47 | 0.29 | 0.91 | 0.63 | 1.33 |
| 4 | SIMD Q3 | -0.21 | -0.59 | 0.17 | 0.81 | 0.55 | 1.19 |
| 4 | SIMD Q4 | -0.11 | -0.49 | 0.27 | 0.90 | 0.61 | 1.31 |
| 4 | SIMD Q5 (Least Depr.) | -0.41 | -0.80 | -0.02 | 0.67 | 0.45 | 0.98 |
| 5 | Shape | -0.01 | -0.02 | -0.00 | NA | NA | NA |
| 5 | Rate | 0.12 | 0.10 | 0.15 | NA | NA | NA |
| 5 | Males (Ref: Females) | 0.18 | 0.05 | 0.31 | 1.19 | 1.05 | 1.36 |
| 5 | SIMD Q2 (Ref: SIMD Q1) | -0.24 | -0.43 | -0.06 | 0.78 | 0.65 | 0.94 |
| 5 | SIMD Q3 | -0.33 | -0.52 | -0.14 | 0.72 | 0.59 | 0.87 |
| 5 | SIMD Q4 | -0.35 | -0.56 | -0.15 | 0.70 | 0.57 | 0.86 |
| 5 | SIMD Q5 (Least Depr.) | -0.42 | -0.64 | -0.20 | 0.66 | 0.53 | 0.82 |
| 6 | Shape | 0.07 | 0.06 | 0.08 | NA | NA | NA |
| 6 | Rate | 0.02 | 0.01 | 0.02 | NA | NA | NA |
| 6 | Males (Ref: Females) | 0.19 | 0.02 | 0.35 | 1.21 | 1.02 | 1.42 |
| 6 | SIMD Q2 (Ref: SIMD Q1) | -0.16 | -0.40 | 0.08 | 0.85 | 0.67 | 1.08 |
| 6 | SIMD Q3 | -0.29 | -0.54 | -0.04 | 0.75 | 0.58 | 0.96 |
| 6 | SIMD Q4 | -0.53 | -0.81 | -0.26 | 0.59 | 0.45 | 0.77 |
| 6 | SIMD Q5 (Least Depr.) | -0.61 | -0.89 | -0.32 | 0.54 | 0.41 | 0.72 |
| 7 | Shape | 0.05 | 0.04 | 0.06 | NA | NA | NA |
| 7 | Rate | 0.04 | 0.03 | 0.06 | NA | NA | NA |
| 7 | Males (Ref: Females) | 0.14 | -0.03 | 0.31 | 1.15 | 0.97 | 1.36 |
| 7 | SIMD Q2 (Ref: SIMD Q1) | -0.18 | -0.43 | 0.06 | 0.83 | 0.65 | 1.06 |
| 7 | SIMD Q3 | -0.17 | -0.42 | 0.08 | 0.85 | 0.66 | 1.09 |
| 7 | SIMD Q4 | -0.06 | -0.32 | 0.20 | 0.94 | 0.72 | 1.22 |
| 7 | SIMD Q5 (Least Depr.) | -0.30 | -0.58 | -0.01 | 0.74 | 0.56 | 0.99 |
