# Supplementary material for: Large socioeconomic gap in period life expectancy and life years spent with complications of diabetes in the Scottish population with type 1 diabetes, 2013–2018
Source: PLoS One. 2022 Aug 11;17(8):e0271110. doi: 10.1371/journal.pone.0271110 (PMC9371295; doi:10.1371/journal.pone.0271110)
Supplement: S7 Table — (DOCX) [file pone.0271110.s007.docx]

**S7 Tables: Overview of the study population from SIMD quintile 1 (most deprived), 2, 3, 4, and 5 (least deprived) at point of study entry including all biomedical information utilized in the sensitivity analysis.**

**S7a Table: Overview of the study population from SIMD quintile 1 (most deprived) at point of study entry including all biomedical information utilized in the sensitivity analysis.**

| Summary | N / Median | Percentage % / (IQR) |
| --- | --- | --- |
| Males | 865 | 56.3% |
| Females | 672 | 43.7% |
| Age (years): Median | 58.9 | (54.0 66.2) |
| Diab. Duration (years): Median | 25.5 | (16.4 36.0) |
| Hba1c mmol/mol: Median | 72.0 | (62.0 84.0) |
| HDL-cholesterol mmol/mol: Median | 1.4 | (1.1 1.8) |
| LDL-cholesterol mmol/mol: Median | 2.2 | (1.7 2.9) |
| Total cholesterol mmol/mol: Median | 4.4 | (3.8 5.1) |
| Systolic BP mmHg: Median | 132.0 | (122.0 143.0) |
| Diastolic BP mmHg: Median | 72.0 | (66.0 80.0) |
| BMI kg/m^2: Median | 27.7 | (24.4 31.3) |
| Ever Smoker | 944 | 61.42% |
| Cardiovascular Disease | 699 | 45.5% |
| Retinopathy/Maculopathy | 305 | 19.8% |
| Chronic Kidney Disease | 621 | 40.4% |
| Diabetic Foot | 178 | 11.6% |
| - Number of Complications - |  |  |
| 0 Complications | 492 | 32.0% |
| 1 Complication | 491 | 31.9% |
| 2 Complications | 388 | 25.2% |
| 3+ Complications | 166 | 10.8% |

**S7b Table: Overview of the study population from SIMD quintile 2 at point of study entry including all biomedical information utilized in the sensitivity analysis.**

| Summary | N / Median | Percentage % / (IQR) |
| --- | --- | --- |
| Males | 1009 | 54.9% |
| Females | 829 | 45.1% |
| Age (years): Median | 59.7 | (54.2 66.4) |
| Diab. Duration (years): Median | 27.0 | (18.0 37.0) |
| Hba1c mmol/mol: Median | 69.9 | (61.0 80.0) |
| HDL-cholesterol mmol/mol: Median | 1.4 | (1.2 1.8) |
| LDL-cholesterol mmol/mol: Median | 2.2 | (1.7 2.8) |
| Total cholesterol mmol/mol: Median | 4.3 | (3.7 5.0) |
| Systolic BP mmHg: Median | 133.5 | (122.0 143.0) |
| Diastolic BP mmHg: Median | 72.0 | (66.0 80.0) |
| BMI kg/m^2: Median | 27.6 | (24.5 30.8) |
| Ever Smoker | 1022 | 55.6% |
| Cardiovascular Disease | 808 | 44.0% |
| Retinopathy/Maculopathy | 321 | 17.5% |
| Chronic Kidney Disease | 781 | 42.5% |
| Diabetic Foot | 190 | 10.3% |
| - Number of Complications - |  |  |
| 0 Complications | 630 | 34.3% |
| 1 Complication | 551 | 30.0% |
| 2 Complications | 460 | 25.0% |
| 3+ Complications | 197 | 10.7% |

**S7c Table: Overview of the study population from SIMD quintile 3 at point of study entry including all biomedical information utilized in the sensitivity analysis.**

| Summary | N / Median | Percentage % / (IQR) |
| --- | --- | --- |
| Males | 1012 | 54.7% |
| Females | 837 | 45.3% |
| Age (years): Median | 59.6 | (54.4 67.1) |
| Diab. Duration (years): Median | 27.0 | (17.8 39.0) |
| Hba1c mmol/mol: Median | 69.0 | (61.0 79.0) |
| HDL-cholesterol mmol/mol: Median | 1.5 | (1.2 1.9) |
| LDL-cholesterol mmol/mol: Median | 2.2 | (1.8 2.8) |
| Total cholesterol mmol/mol: Median | 4.3 | (3.8 5.0) |
| Systolic BP mmHg: Median | 134.5 | (125.0 145.0) |
| Diastolic BP mmHg: Median | 73.0 | (67.0 80.0) |
| BMI kg/m^2: Median | 27.5 | (24.5 30.8) |
| Ever Smoker | 917 | 49.59% |
| Cardiovascular Disease | 723 | 39.1% |
| Retinopathy/Maculopathy | 294 | 15.9% |
| Chronic Kidney Disease | 742 | 40.1% |
| Diabetic Foot | 165 | 8.9% |
| - Number of Complications - |  |  |
| 0 Complications | 710 | 38.4% |
| 1 Complication | 552 | 29.9% |
| 2 Complications | 414 | 22.4% |
| 3+ Complications | 173 | 9.4% |

**S7d Table: Overview of the study population from SIMD quintile 4 at point of study entry including all biomedical information utilized in the sensitivity analysis.**

| Summary | N / Median | Percentage % / (IQR) |
| --- | --- | --- |
| Males | 888 | 54.9% |
| Females | 731 | 45.1% |
| Age (years): Median | 59.7 | (54.5 67.5) |
| Diab. Duration (years): Median | 27.7 | (17.5 39.0) |
| Hba1c mmol/mol: Median | 68.0 | (60.0 77.0) |
| HDL-cholesterol mmol/mol: Median | 1.6 | (1.2 1.9) |
| LDL-cholesterol mmol/mol: Median | 2.2 | (1.8 2.8) |
| Total cholesterol mmol/mol: Median | 4.4 | (3.8 5.0) |
| Systolic BP mmHg: Median | 135.0 | (124.0 145.0) |
| Diastolic BP mmHg: Median | 73.0 | (66.0 80.0) |
| BMI kg/m^2: Median | 27.0 | (24.2 30.4) |
| Ever Smoker | 717 | 44.29% |
| Cardiovascular Disease | 581 | 35.9% |
| Retinopathy/Maculopathy | 264 | 16.3% |
| Chronic Kidney Disease | 625 | 38.6% |
| Diabetic Foot | 128 | 7.9% |
| - Number of Complications - |  |  |
| 0 Complications | 644 | 39.8% |
| 1 Complication | 515 | 31.8% |
| 2 Complications | 323 | 19.9% |
| 3+ Complications | 137 | 8.5% |

**S7e Table: Overview of the study population from SIMD quintile 5 (least deprived) at point of study entry including all biomedical information utilized in the sensitivity analysis.**

| Summary | N / Median | Percentage % / (IQR) |
| --- | --- | --- |
| Males | 980 | 56.1% |
| Females | 768 | 43.9% |
| Age (years): Median | 60.4 | (54.6 67.3) |
| Diab. Duration (years): Median | 28.0 | (18.0 40.0) |
| Hba1c mmol/mol: Median | 67.0 | (58.0 76.0) |
| HDL-cholesterol mmol/mol: Median | 1.6 | (1.3 1.9) |
| LDL-cholesterol mmol/mol: Median | 2.2 | (1.8 2.8) |
| Total cholesterol mmol/mol: Median | 4.4 | (3.8 5.1) |
| Systolic BP mmHg: Median | 134.0 | (124.0 144.0) |
| Diastolic BP mmHg: Median | 73.0 | (66.0 80.0) |
| BMI kg/m^2: Median | 26.8 | (24.1 30.0) |
| Ever Smoker | 665 | 38.04% |
| Cardiovascular Disease | 552 | 31.6% |
| Retinopathy/Maculopathy | 219 | 12.5% |
| Chronic Kidney Disease | 601 | 34.4% |
| Diabetic Foot | 112 | 6.4% |
| - Number of Complications - |  |  |
| 0 Complications | 798 | 45.6% |
| 1 Complication | 548 | 31.4% |
| 2 Complications | 285 | 16.3% |
| 3+ Complications | 117 | 6.7% |
