# Supplementary material for: Large socioeconomic gap in period life expectancy and life years spent with complications of diabetes in the Scottish population with type 1 diabetes, 2013–2018
Source: PLoS One. 2022 Aug 11;17(8):e0271110. doi: 10.1371/journal.pone.0271110 (PMC9371295; doi:10.1371/journal.pone.0271110)
Supplement: S8 Table — (DOCX) [file pone.0271110.s008.docx]

**S8 Table: Comparison of Hazard Ratios (HR) for sex and SIMD quintile from the utilized transition-specific models of set 2 with HR obtained from set 3.**

**Note:** Set 3 represents a corresponding model which – in addition to the covariates sex and SIMD quintile – includes information on smoking status, Hba1c, BMI, diastolic BP, systolic BP, total cholesterol, low-density lipoprotein-cholesterols, and high-density lipoprotein-cholesterols. Transitions align directly to Fig 1 - Panel (B).

| Transition | Parameter | HR | L95 | H95 | HR.Comp | L95.Comp | H95.Comp |
| --- | --- | --- | --- | --- | --- | --- | --- |
| 1 | Males (Ref: Females) | 1.20 | 1.06 | 1.35 | 1.21 | 1.06 | 1.37 |
| 1 | SIMD Q2 (Ref: SIMD Q1) | 0.83 | 0.69 | 1.00 | 0.86 | 0.71 | 1.04 |
| 1 | SIMD Q3 | 0.73 | 0.60 | 0.87 | 0.79 | 0.65 | 0.95 |
| 1 | SIMD Q4 | 0.69 | 0.57 | 0.84 | 0.78 | 0.65 | 0.95 |
| 1 | SIMD Q5 (Least Depr.) | 0.63 | 0.52 | 0.75 | 0.73 | 0.61 | 0.89 |
| 2 | Males (Ref: Females) | 1.62 | 1.07 | 2.45 | 1.57 | 1.01 | 2.45 |
| 2 | SIMD Q2 (Ref: SIMD Q1) | 0.55 | 0.32 | 0.94 | 0.58 | 0.34 | 0.99 |
| 2 | SIMD Q3 | 0.49 | 0.29 | 0.84 | 0.57 | 0.33 | 0.97 |
| 2 | SIMD Q4 | 0.23 | 0.11 | 0.45 | 0.27 | 0.14 | 0.55 |
| 2 | SIMD Q5 (Least Depr.) | 0.29 | 0.16 | 0.52 | 0.36 | 0.20 | 0.66 |
| 3 | Males (Ref: Females) | 1.15 | 1.03 | 1.28 | 1.12 | 1.00 | 1.26 |
| 3 | SIMD Q2 (Ref: SIMD Q1) | 0.95 | 0.80 | 1.11 | 0.95 | 0.81 | 1.12 |
| 3 | SIMD Q3 | 0.84 | 0.72 | 1.00 | 0.87 | 0.73 | 1.02 |
| 3 | SIMD Q4 | 0.79 | 0.67 | 0.94 | 0.84 | 0.71 | 1.00 |
| 3 | SIMD Q5 (Least Depr.) | 0.67 | 0.56 | 0.80 | 0.73 | 0.61 | 0.87 |
| 4 | Males (Ref: Females) | 1.65 | 1.29 | 2.12 | 1.44 | 1.10 | 1.88 |
| 4 | SIMD Q2 (Ref: SIMD Q1) | 0.91 | 0.63 | 1.33 | 0.94 | 0.65 | 1.38 |
| 4 | SIMD Q3 | 0.81 | 0.55 | 1.19 | 0.93 | 0.64 | 1.37 |
| 4 | SIMD Q4 | 0.90 | 0.61 | 1.31 | 1.06 | 0.72 | 1.56 |
| 4 | SIMD Q5 (Least Depr.) | 0.67 | 0.45 | 0.98 | 0.79 | 0.53 | 1.17 |
| 5 | Males (Ref: Females) | 1.19 | 1.05 | 1.36 | 1.22 | 1.06 | 1.40 |
| 5 | SIMD Q2 (Ref: SIMD Q1) | 0.78 | 0.65 | 0.94 | 0.83 | 0.69 | 0.99 |
| 5 | SIMD Q3 | 0.72 | 0.59 | 0.87 | 0.77 | 0.63 | 0.93 |
| 5 | SIMD Q4 | 0.70 | 0.57 | 0.86 | 0.77 | 0.62 | 0.95 |
| 5 | SIMD Q5 (Least Depr.) | 0.66 | 0.53 | 0.82 | 0.72 | 0.58 | 0.89 |
| 6 | Males (Ref: Females) | 1.21 | 1.02 | 1.42 | 0.98 | 0.82 | 1.17 |
| 6 | SIMD Q2 (Ref: SIMD Q1) | 0.85 | 0.67 | 1.08 | 0.90 | 0.71 | 1.14 |
| 6 | SIMD Q3 | 0.75 | 0.58 | 0.96 | 0.83 | 0.65 | 1.07 |
| 6 | SIMD Q4 | 0.59 | 0.45 | 0.77 | 0.62 | 0.47 | 0.82 |
| 6 | SIMD Q5 (Least Depr.) | 0.54 | 0.41 | 0.72 | 0.60 | 0.45 | 0.80 |
| 7 | Males (Ref: Females) | 1.15 | 0.97 | 1.36 | 1.00 | 0.84 | 1.20 |
| 7 | SIMD Q2 (Ref: SIMD Q1) | 0.83 | 0.65 | 1.06 | 0.83 | 0.65 | 1.06 |
| 7 | SIMD Q3 | 0.85 | 0.66 | 1.09 | 0.88 | 0.69 | 1.14 |
| 7 | SIMD Q4 | 0.94 | 0.72 | 1.22 | 0.98 | 0.75 | 1.27 |
| 7 | SIMD Q5 (Least Depr.) | 0.74 | 0.56 | 0.99 | 0.78 | 0.58 | 1.04 |
