# Supplementary material for: Large socioeconomic gap in period life expectancy and life years spent with complications of diabetes in the Scottish population with type 1 diabetes, 2013–2018
Source: PLoS One. 2022 Aug 11;17(8):e0271110. doi: 10.1371/journal.pone.0271110 (PMC9371295; doi:10.1371/journal.pone.0271110)
Supplement: S1 Fig — (DOCX) [file pone.0271110.s009.docx]

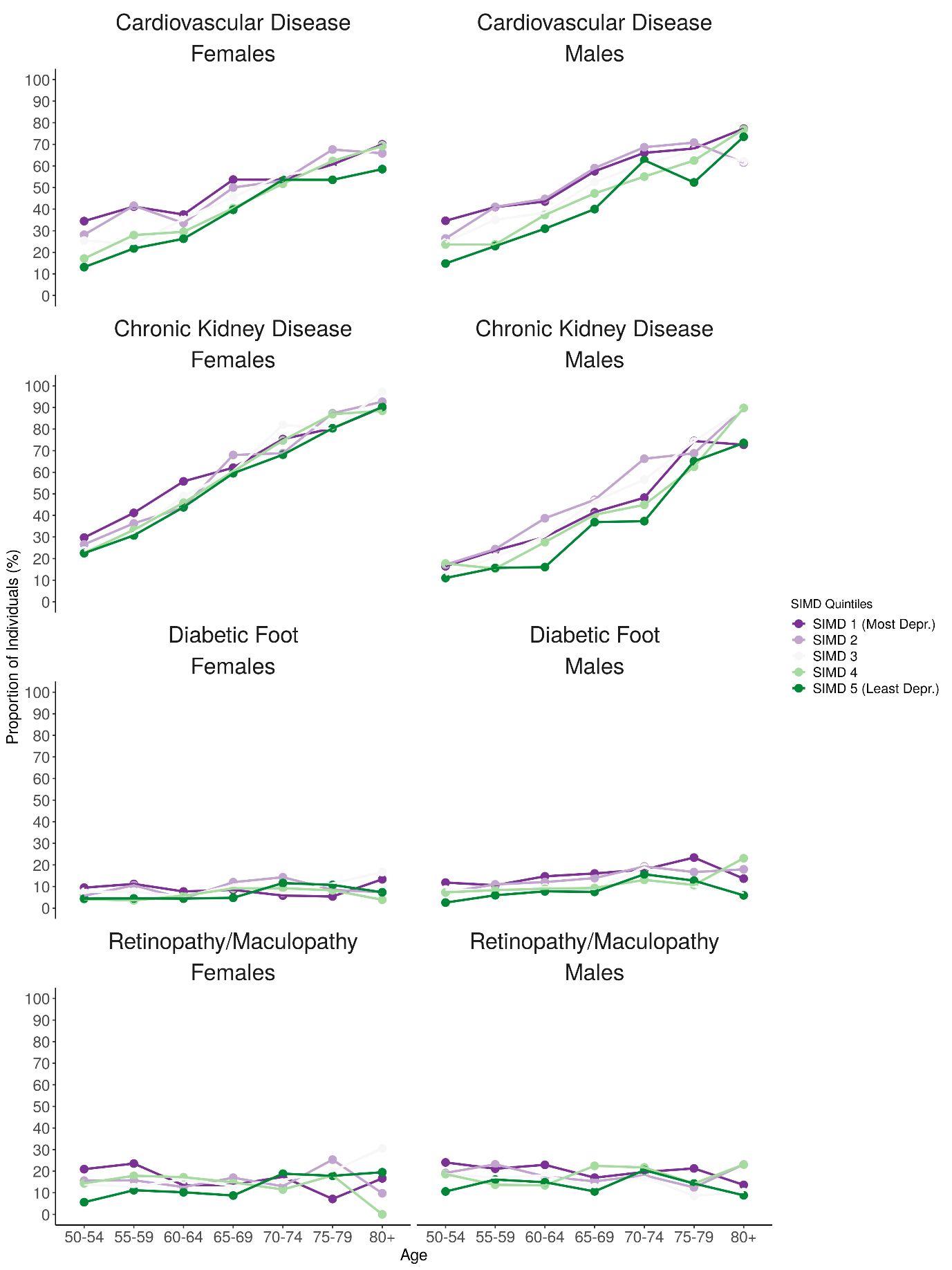


**S1 Fig: Prevalence of the four studied complications retinopathy/maculopathy, cardiovascular disease, chronic kidney disease, and diabetic foot within the study population at point of study entry, separately for males and females and by SIMD quintile.**
