# Supplementary material for: Large socioeconomic gap in period life expectancy and life years spent with complications of diabetes in the Scottish population with type 1 diabetes, 2013–2018
Source: PLoS One. 2022 Aug 11;17(8):e0271110. doi: 10.1371/journal.pone.0271110 (PMC9371295; doi:10.1371/journal.pone.0271110)
Supplement: S2 Fig — (DOCX) [file pone.0271110.s010.docx]

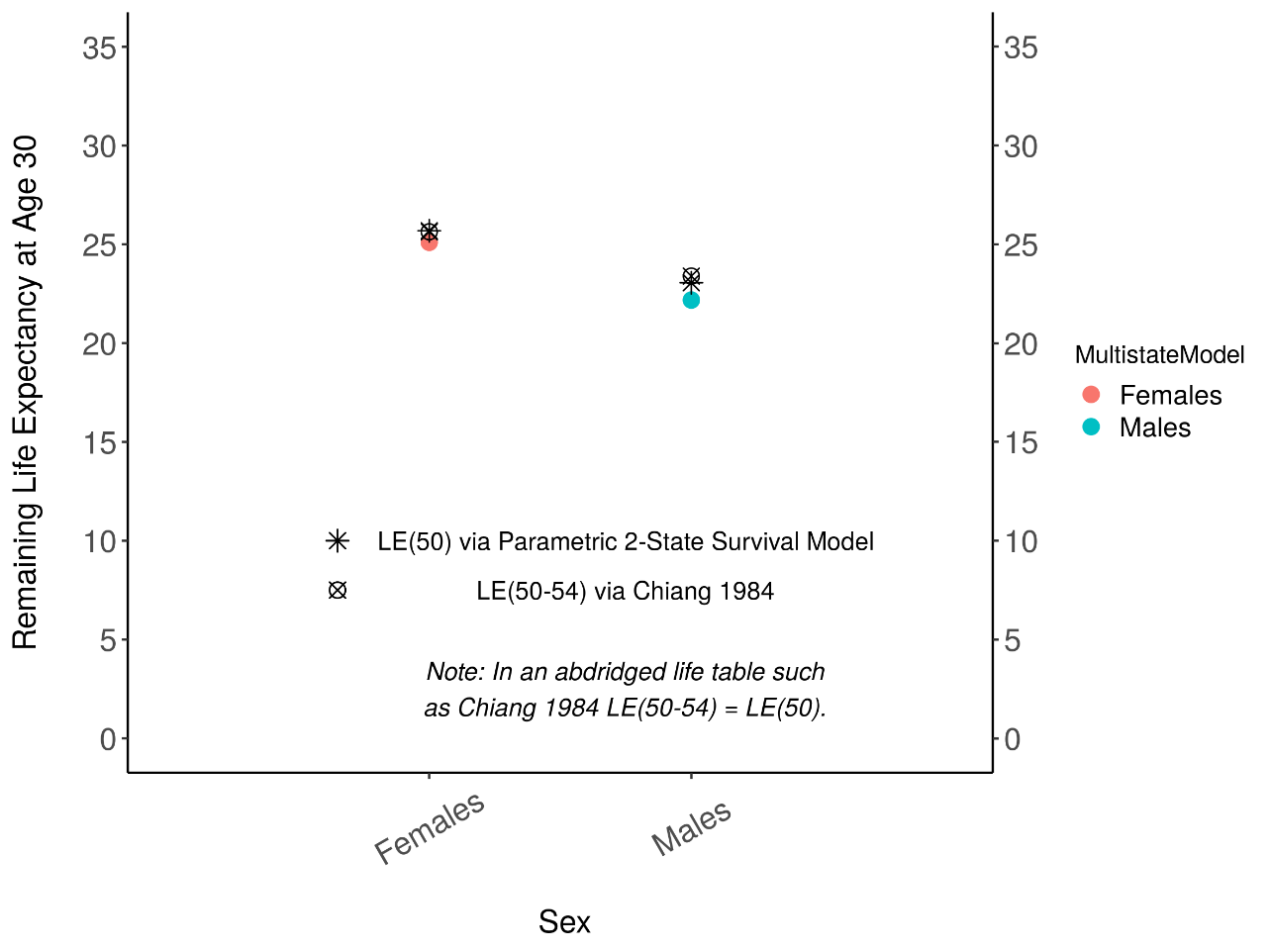


**S2 Fig:** **Comparison of estimates for Life Expectancy at age 50 for males and females obtained from the multistate survival model (main findings presented in the paper) with corresponding LE estimates we derived using the Chiang (1984) life table method and parametric two-state survival models.**
