# Supplementary material for: Large socioeconomic gap in period life expectancy and life years spent with complications of diabetes in the Scottish population with type 1 diabetes, 2013–2018
Source: PLoS One. 2022 Aug 11;17(8):e0271110. doi: 10.1371/journal.pone.0271110 (PMC9371295; doi:10.1371/journal.pone.0271110)
Supplement: S1 Text — (DOCX) [file pone.0271110.s012.docx]

**S1 Text: R-Code example demonstrating the estimation of total- and complication-adjusted LE.**

In this example, we demonstrate the procedure of deriving state expectancy estimates. Unfortunately, we are not allowed to share the underlying data and related data objects due to data confidentiality. However, interested researchers can contact the corresponding author and/or apply for data access. The following code does not meet the minimum requirements of a minimum working example. However, we present all results along the way to ensure a maximum amount of transparency.

In this example, we replicate the results for all men and women shown in Figure 4. To use this code for your own example, you will be required to adapt it to your data structure. This may require changes in the matrix of allowed transitions, the functional form of the parametric models etc. In addition, you need to load the libraries "data.table", "flexsurv", and "mstate".

Theoretically, all calculations presented in Figure 4 can be reproduced correctly by hand using the following two tables: "R.state.dist.sex" - distribution of start states and "R.results.list" - matrix of start-state-specific state expectancies. For example: Females, years spent with 1 complication = (7.527259 * 0.5816697) + (11.30076 * 0.2681488) = 7.408663 years. Comparison with results from Figure 4 in the main manuscript: 7.4 years

# ----------------------- #

### (1) DEFINITIONS ###

# age we focus on

R.age.center <- 50

# maximum theoretical life span to solve integral

R.max.age <- 110

# Allowed Transitions Matrix

# Corresponds to Figure 1 - Panel B in the Main Manuscript

R.tmat <- mstate::transMat(x = list(c(2,5),

c(3,5),

c(4,5),

c(5),

c()),

names = c("0C", "1C", "2C", "3C", "Death"))

print(R.tmat)

#to

#from 0C 1C 2C 3C Death

#0C NA 1 NA NA 2

#1C NA NA 3 NA 4

#2C NA NA NA 5 6

#3C NA NA NA NA 7

#Death NA NA NA NA NA

# Number of possible transitions #

R.no.of.trans <- sum(!is.na(R.tmat))

# Formula for Parametric Model

R.formula.sex <- noquote("Surv(Tstart, Tstop, status) ~ men")

# Start State Distribution = start states at ages 45-54

# corresponds to S4 Table in the Supplementary Material

# men = 1 = men / men = 0 = women

# V1 = 0 Compl. / V2 = 1 Complc / V3 = 2 Compl. / V4 = 3+ Compl

R.state.dist.sex <- state.dist.sex

print(R.state.dist.sex)

# men V1 V2 V3 V4

# 1: 0 0.5816697 0.2681488 0.1016334 0.04809437

# 2: 1 0.5948869 0.2477876 0.1065225 0.05047525

# ----------------------- #

### (2) ESTIMATE PARAMETRIC MODEL FOR EACH TRANSITION ###

R.model.list <- vector(no.of.trans, mode="list")

for (i in 1:R.no.of.trans) {

# markov model: clock forward - no dependency on time spend in previous states #

R.model.list[[i]] <- flexsurv::flexsurvreg(as.formula(noquote(R.formula.sex)),

subset=(trans==i), data = analysis.dt, dist = "gompertz")

}

# regression results are presented in S5 Table

# Transition 1:

# data mean est L95% U95% se exp(est) L95% U95%

# men1 0.59927 0.17799 0.05744 0.29855 0.06151 1.19481 1.05912 1.34790

# Transition 2:

# data mean est L95% U95% se exp(est) L95% U95%

# men1 0.59927 0.46760 0.05364 0.88157 0.21121 1.59617 1.05510 2.41468

# ...

# ----------------------- #

### (3) DERIVE START-STATE SPECIFIC STATE EXPECTANCIES ###

# create empty new data table

R.new.data <- data.table(men=as.factor(c(0,1)))

setkey(R.new.data, men)

# make a list to store predictions

R.new.data.list <- vector(dim(R.new.data)[1], mode="list")

for (i in 1:dim(R.new.data)[1]) {

R.new.data.list[[i]] <- data.table(

rbind(R.new.data[i],R.new.data[i],R.new.data[i],R.new.data[i],

R.new.data[i],R.new.data[i],R.new.data[i]),

trans=c(1,2,3,4,5,6,7))

}

# make predictions and store results in a list

R.results.list <- vector(dim(R.new.data)[1], mode="list")

# predict conditional state expectancies for all men and women using

for (i in 1:dim(R.new.data)[1]){

R.results.list[[i]] <- flexsurv::totlos.fs(R.model.list,

trans=R.tmat, t=R.max.age-R.age.center, newdata=R.new.data.list[[i]],

ci=FALSE, sing.inf = 1e+100)

}

# print predicted matrix of start-state-specific state expectancies

print(R.results.list)

# [[1]]

# [,1] [,2] [,3] [,4] [,5]

# [1,] 13.41148 7.527259 4.962326 2.454006 31.64493

# [2,] 0.00000 11.300756 6.803981 4.793552 37.10171

# [3,] 0.00000 0.000000 8.711769 8.824978 42.46325

# [4,] 0.00000 0.000000 0.000000 14.271944 45.72806

# [5,] 0.00000 0.000000 0.000000 0.000000 60.00000

# [[2]]

# [,1] [,2] [,3] [,4] [,5]

# [1,] 11.67955 6.579068 4.203362 2.485567 35.05245

# [2,] 0.00000 9.831492 5.702548 4.666433 39.79953

# [3,] 0.00000 0.000000 7.369687 8.413895 44.21642

# [4,] 0.00000 0.000000 0.000000 12.974333 47.02567

# [5,] 0.00000 0.000000 0.000000 0.000000 60.00000

# ----------------------- #

### (4) ESTIMATE POPULATION-LEVEL STATE EXPECTANCIES ###

# Total Life Excpectancy #

R.life.expectancy.total <- rep(99, dim(R.new.data)[1])

for (i in 1:dim(new.data)[1]) {

R.life.expectancy.total[i] <-

(R.results.list[[i]][1,1] * R.state.dist.sex$V1[i]) + # healthy

(R.results.list[[i]][1,2] * R.state.dist.sex$V1[i]) + # 1 Complication

(R.results.list[[i]][2,2] * R.state.dist.sex$V2[i]) + #

(R.results.list[[i]][1,3] * R.state.dist.sex$V1[i]) + # 2 Complications

(R.results.list[[i]][2,3] * R.state.dist.sex$V2[i]) + #

(R.results.list[[i]][3,3] * R.state.dist.sex$V3[i]) + #

(R.results.list[[i]][1,4] * R.state.dist.sex$V1[i]) + # 3+ Complications

(R.results.list[[i]][2,4] * R.state.dist.sex$V2[i]) + #

(R.results.list[[i]][3,4] * R.state.dist.sex$V3[i]) + #

(R.results.list[[i]][4,4] * R.state.dist.sex$V4[i]) #

}

R.new.data.1 <- copy(R.new.data)

R.new.data.1[, LE := R.life.expectancy.total][,

type := as.factor("Total")]

# ----------------------- #

# Time with No Complications #

R.life.expectancy.healthy <- rep(99, dim(R.new.data)[1])

for (i in 1:dim(R.new.data)[1]) {

R.life.expectancy.healthy[i] <- R.results.list[[i]][1,1] *

R.state.dist.sex$V1[i]

}

R.new.data.2 <- copy(R.new.data)

R.new.data.2[, LE := R.life.expectancy.healthy][,

type := as.factor("0 Complications")]

# ----------------------- #

# 1 Complication

R.life.expectancy.1c <- rep(99, dim(R.new.data)[1])

for (i in 1:dim(R.new.data)[1]) {

R.life.expectancy.1c[i] <-

(results.list[[i]][1,2] * R.state.dist.sex$V1[i]) +

(results.list[[i]][2,2] * R.state.dist.sex$V2[i])

}

R.new.data.3 <- copy(R.new.data)

R.new.data.3[, LE := R.life.expectancy.1c][,

type := as.factor("1 Complication")]

# ----------------------- #

# 2 Complications

R.life.expectancy.2c <- rep(99, dim(R.new.data)[1])

for (i in 1:dim(R.new.data)[1]) {

R.life.expectancy.2c[i] <-

(R.results.list[[i]][1,3] * R.state.dist.sex$V1[i]) +

(R.results.list[[i]][2,3] * R.state.dist.sex$V2[i]) +

(R.results.list[[i]][3,3] * R.state.dist.sex$V3[i])

}

R.new.data.4 <- copy(R.new.data)

R.new.data.4[, LE := R.life.expectancy.2c][,

type := as.factor("2 Complications")]

# ----------------------- #

# 3+ Complications

R.life.expectancy.3c <- rep(99, dim(R.new.data)[1])

for (i in 1:dim(new.data)[1]) {

R.life.expectancy.3c[i] <-

(R.results.list[[i]][1,4] * R.state.dist.sex$V1[i]) +

(R.results.list[[i]][2,4] * R.state.dist.sex$V2[i]) +

(R.results.list[[i]][3,4] * R.state.dist.sex$V3[i]) +

(R.results.list[[i]][4,4] * R.state.dist.sex$V4[i])

}

R.new.data.5 <- copy(R.new.data)

R.new.data.5[, LE := R.life.expectancy.3c][,

type := as.factor("3+ Complications")]

# ------------------------#

### (5) Combine and Examine Results ###

# combine and examine result #

R.result <- rbind(R.new.data.1, R.new.data.2, R.new.data.3,

R.new.data.4, R.new.data.5)

print(R.result)

# men = 1 = men / men = 0 = women

# men LE type

# 1: 0 25.102153 Total

# 2: 1 22.182585 Total

# 3: 0 7.801051 0 Complications

# 4: 1 6.948011 0 Complications

# 5: 0 7.408657 1 Complication

# 6: 1 6.349878 1 Complication

# 7: 0 5.596321 2 Complications

# 8: 1 4.698583 2 Complications

# 9: 0 4.296119 3+ Complications

# 10: 1 4.186067 3+ Complications

# these values correspond directly to Figure 4 of the main manuscript

# ------------------------#
