# Supplementary material for: Large socioeconomic gap in period life expectancy and life years spent with complications of diabetes in the Scottish population with type 1 diabetes, 2013–2018
Source: PLoS One. 2022 Aug 11;17(8):e0271110. doi: 10.1371/journal.pone.0271110 (PMC9371295; doi:10.1371/journal.pone.0271110)
Supplement: Attachment — Submitted filename: 2 Respose to Reviewers.docx [file pone.0271110.s014.docx]

**Response to Reviewers**

We would like to thank the expert reviewer and the academic editor for the positive, constructive, and helpful feedback. We have summarised the changes we have made below. We provide a clear justification in response to the few comments we have not been able to incorporate.

**Comments from the Academic Editor**

1. **Provide a more equilibrated view on the available literature in the "Introduction". You have stated "Estimates of LE [4–6] and health-adjusted LE [7, 8] are available for the population with type 1 diabetes". However, after that the authors unexpectedly focused on the discussion of shortages of different studies presented by the references 9 and further. To provide equilibrated view on the literature, please discuss the findings described in the references 4-8 in this section of the "Introduction".**

Thank you very much for pointing this out. We have substantially revised the introduction. We now provide a detailed summary on all previous findings. The revision of the introduction now provides a much stronger background for our study and strengthens the demographic focus of this paper. Your comment prompted us to further strengthen the rationale and importance of our study.

2. **The phrase "As is typically observed in general populations, we hypothesized that LE would be higher among females than males". is unclear. What are the consequences of this hypothesis, and what for it has been introduced if you have real-world data that do not need any a priori hypotheses for the analysis? In general, instead of a description of anticipated findings, please describe in the "Introduction" the major goals of your analysis and endpoints.**

We agree with the reviewer and now state clearly that our goal was to estimate LE for males and females of the Scottish population with type 1 diabetes at age 50 for different socioeconomic groups and to estimate how many subsequent years of life were spent with and without the most common complications of diabetes.

In the revised the introduction, we now provide a much clearer context for our goal as well as our hypotheses. The editorial guidelines^[1]^ of PLOS ONE advice authors of cohort studies to comply with research checklists such as STROBE (Vandenbroucke et al. 2007), which asks authors to specify aims and pre-specified hypotheses in the introduction. We therefore decided to not exclude our hypotheses as they provide important guidance for the reader. We discuss these hypotheses in the discussion of our manuscript in light of the findings of our analysis.

3. **The authors described major steps in the analysis and supported them by the references to the previous publications in the "Data" section. However, this is not enough. Please provide enough details to make the self-sufficient description of the study population and data organisation. The authors could provide in the Supplement the exact list of drug prescriptions and clinical information on the type of diabetes used for the selection of study sample. As of one of the major points of interest, please describe in details how you have identified the DM type (1 or 2) that is a difficult task the cohort of persons 50+ years old.**

Thank you for requesting further details. We have now included more information on the algorithm used to distinguish between individuals with type 1 diabetes from other forms of diabetes in the SCI Diabetes data base. Several elements of the database, such as data acquisition, data linkage, data cleaning and the type-of-diabetes-algorithm, have been described in more detail across different studies. We now provide references to the studies describing these elements in more detail. Unfortunately, there is currently no published cohort profile study. The editor’s comment has highlighted to our research group, the increasing importance of publishing a cohort profile study, which is currently work in progress.

4. **Regarding the cause of death analysis, please indicate whether you have used the multiple cause of death database available in Scotland, or classified a cause of death based on a single-reason approach.**

Thank you for asking for clarification. Mortality was captured as all-cause mortality only.

No analysis presented in the paper is an analysis of causes of death or required a differentiation by cause of death. We have provided clarification in the paper.

5. **Please indicate more details about "sociodemographic data and information on health status were obtained and continuously updated using a long-format survival table capturing 30-day slices of time". Please indicate how you managed the analysis in the case of a person changed the residence from more prominent to less prominent social deprivation area defined by SIMD.**

Thank you for showing an interest in these important points.

In the methods and materials section, we have now described how the 30-day slices of time were created.

New releases of the Scottish Index of Multiple Deprivation (SIMD) are issued every 4 years by the Scottish Government. Previous releases have been published for 2009, 2012, 2016, and 2020. However, the 2012 and 2016 SIMD releases are the only ones we have incorporated into the SCI diabetes database. We used the 2016 release consistently throughout the entire study period. This is consistent with the recommendations made by the Information Services Division (ISD) of NHS Scotland who are responsible for estimating the SIMD.^[2]^ By using the 2016 release, we comply with the recommendations for working with the SIMD longitudinally: (1) we have used the most appropriate release for the study period 2013-2018 and (2) we have used one release consistently for all individuals throughout the entire study period. This response is also relevant to comment #1 made by the expert reviewer.

6. **The reference 20 intended to describe the calculation of SIMD does not contain enough bibliographic details to obtain the document and familiarise with the methodology of SIMD calculation.**

Thank you for noticing our oversight. We have now included the correct title of the publication and a link to the online source.

7. **Please describe in details the ICD codes (or other codes or information classification) that have been used to obtain information about the four diabetes-related complications: retinopathy/maculopathy, cardiovascular disease (CVD), nephropathy, and neuropathy.**

Thank you for requesting more detail. We have revised S1 Table and its caption accordingly. We have now included information on the sources of all ICD codes and screening results.

8. **Regarding nephropathy, the author indicated only low eGFR and renal replacement therapy among the criteria. However, the first signs of diabetic nephropathy are presented by elevated albuminuria (or ACR ratio), while decreasing eGFR is rather late sign of diabetic nephropathy. Please describe whether the information about albuminuria is available in the registry you used. Please also indicate which eGFR equation has been used, and most importantly - whether the confirmation of initial abnormally low eGFR has been considered to correctly classify a patient. Please indicate how many patients had low GFR only, and how many received renal replacement therapy, instead of merging these highly heterogeneous conditions together.**

Thank you very much for this excellent comment which we entirely agree with. We now state in the ‘Methods and materials section’ that we utilized the CKD-EPI equation (Levey et al. 2009) and state the number of individuals in receipt of renal replacement therapy in the results section.

We have focussed on reduced eGFR (specifically: chronic kidney disease stage 3 or worse) rather than albuminuria for two reasons. First of all, it has been pointed out that ACR lacks specificity and sensitivity for progressive decline in eGFR (Krolewski 2015). For example, a poor positive predictive value was reported, with only about a third of those with microalbuminuria having progressive renal function decline (Krolewski 2015). Furthermore, albumin excretion also had low sensitivity in that only about half of those with progressive renal function decline were albuminuric (Krolewski 2015). For Scotland, we have found very similar results very recently: The majority (59%) of those with chronic kidney disease stages G3–G5 did not have albuminuria on the day of recruitment or previously (Colombo et al. 2020)

The second reason we have focussed on eGFR is that it is measured very regularly in our cohort; typically every year with 86% of those with type 1 diabetes having an eGFR measurement in the past year, while only 62% had ACR measured.^[3]^. We have rephrased throughout the manuscript that we are referring to CKD rather than an albuminuria based concept.

Our current standard data cleaning algorithm for eGFR is able to identify single acute transient drops in eGFR. These acute transient drops were filtered and are not considered when defining CKD.

9. **Considering the importance of the state space and all seven distinct transitions for understanding the results of the analysis, please move the details provided in the Figure S1 to the main manuscript. Please explain how "transient states can be entered and left" if previously it has been stated that the complications were considered irreversible, and confirmed this in a couple of sentences later "Transitions back to previous states were not allowed as we assumed complications to be irreversible"? Please explain the meaning of the phrase "we did not allow transitions that skipped one or more transient states".**

We fully agree with the editor regarding Figure S1, thank you very much for this important comment. We have moved Figure S1 into the main manuscript. We can see why there may have been some confusion in regards to our description of the transient states. We now clarify that transient states can only ever be left when individuals were diagnosed with a new complication or have died. Transitions back to previous states were not allowed. We assumed that the onset of complications and the process of accumulating complications was an irreversible process.

We have now also clarified which transitions were not allowed. These revisions can be found in the “Methods and materials / Multistate approach” section. We furthermore clarified explicitly that the following transitions were not allowed in our modelling approach due to a very small number of events (leading to model non-convergence): State 1 – State 3, State 1 –State 4, State 2 – State 4.

10. **At line 174, the authors indicated "We estimated two sets of seven parametric models.", but later described only two sets, with reference to the Supplementary containing description of 7 models. Please describe 7 models in brief also in the main manuscript.**

Thank you very much for this comment. We now detail explicitly why two sets of models were required in the ‘Methods and materials’ section. We have now also pointed out more clearly that the functional form within each set is identical (set 1: ‘sex’ for all seven models / set 2: ‘sex+simd’ for all seven models). This prompted us to refer to the set of 7 models discussed in the sensitivity analysis as a third set of models throughout the manuscript as it is a separate entity and not part of the main analysis.

11. **I could not find the information on how you obtained the "weight of state j".**

Thank you for pointing this out. We have revised the paragraph ‘Estimation of life expectancy and health-adjusted life expectancy’ accordingly. We clarified that the weight of state j corresponds to the prevalence (in other words: relative frequency) of individuals aged 45-54 with either no, 1, 2, or 3+ complications on 01 January 2013 (van den Hout et al. 2019). We have ensured that this terminology has been used consistently throughout the manuscript and the supplementary material.

12. **Please provide appropriate citations for the R packages used in the analysis, the PLOS ONE has no limits to the reference number.**

Thank you for pointing out our oversight. We have now included the exact reference for the utilized R packages ‘Amelia II’, ‘flexsurv’, and for the statistical software ‘R’ itself.

13. **The described approach is interesting and sophisticated. Please provide an example of the calculations performed for several patients included in the analysis (or several patients based on generated input data).**

Thank you very much for this positive comment. The primary aim of our paper was to obtain population-level metrics (life expectancy/ health-adjusted life expectancy) providing intuitive summary indicators for real-world population subgroups. It is not the aim of our paper to provide detailed predictions for individual patients based on a set of specific clinical characteristics. Such analyses are possible – but would require a different study design and methodological approach – and have already been carried out using data for clinical trial population as cited in the introduction.

One more technical note: For individual-level calculations to be meaningful, stronger assumptions are required than for population-level quantities. Typically, the Markov assumption is likely to be violated to some extent at the individual level. While problematic for individual-level estimates, this violation was shown to not bias estimates of state expectancies (Datta and Satten 2001). This means that presenting individual-level calculations even just for illustration might be problematic and potentially misleading.

14. **It is unclear why the authors did not include serum creatinine (and urinary albumin if available) among the list of biochemical and clinical parameters (HbA1c, total cholesterol, high-density lipoprotein-cholesterol, low-density lipoprotein-cholesterol, etc.) used for the modelling.**

We assume that this comment was made about the third set of models, which is part of a sensitivity analysis. The primary aim of this sensitivity analysis was to identify whether the covariate effects of ‘sex’ and ‘SIMD quintile’ in the transition-specific models would disappear, when including information on a number of health risk factors. The sensitivity analysis models do not reflect every single potential health indicator available and was not conceptualized to maximize model performance. As the effects of sex and SIMD remain, this suggests that risk factors included in the sensitivity analysis do not fully explain inequalities in LE and health-adjusted LE. As population-level metrics and socioeconomic inequalities are the focus of this paper, as opposed to detailed predictions based on clinical parameters, we have chosen not to include any additional parameters in the already extensive sensitivity analysis.

15. **Please use one decimal sign for the vast majority of parameters during the presentation of descriptive statistics (individuals mean age of 61.40 has no too much meaning compared to 61.4). Please use Me and IQR for the description of diabetes duration and other variables with not normal distribution.**

We now provide one decimal when presenting descriptive results. We decided to stick with two decimals for presenting our model parameters and LE estimates. We fully agree with the editor as all metric variables for which we previously presented the mean and SD in the table are not normally distributed. For age, diabetes duration, and the length of follow-up period, we now present the Median and IQR. As 6,769 out of 8,591 individuals were followed for the entire 6-year period, the median was 6.0 (IQR of 6.0 6-0) years. We have therefore decided to provide information on the mean in the table 1 caption.

16. **If only diabetic foot has been considered as a neurological complication, please use "diabetic foot "instead of "Neuropathy".**

This is a very good point. We have corrected this throughout the manuscript.

17. **Please move the table(s) describing transitions in the main manuscript instead of pacing it in the Supplementary only.**

Thank you very much for this suggestion. We have now included the most relevant table describing the transitions into the main manuscript as Table 2.

18. **Considering the most and least deprived quantiles, the authors have found almost 4-times difference in mortality rates expressed in person-years for males, but only 1.5-fold difference among females. However, the difference in the LE was not so prominent and was about 8 years for both males and females. Please explain the contrast results between PY-based mortality rates and LE metrics.**

Thank you for showing interest in the different mortality metrics used. We assume that your comment refers to Figure 3 of the revision, which shows age-specific mortality rates observed in the study population.

Remaining life expectancy was calculated at age 50 and included ages 50 to 110. Differences in age-specific mortality rates (i.e. age group 50-54) cannot be directly compared with remaining life expectancy for this age group.

There is no direct relationship between the magnitude of differences in life expectancy at birth and the crude death rate which includes all ages. This also holds when comparing life expectancy with age-standardized mortality rates.

This can be illustrated by using data from the World Bank^[4]^ online tool to complete the example from a demography text book (Preston et al. 2001). The table below shows mortality metrics for females in Sweden and females in Kazakhstan in 1992. The example illustrates that direct comparisons of the magnitude of difference are not possible between different mortality metrics. We would therefore not be able to make any statements about the magnitude of differences between the mortality metrics we used.

| **Indicators*** | **Sweden** | **Kazakhstan** | **Absolute Diff.** | **Relative Diff.** |
| --- | --- | --- | --- | --- |
| Life Expectancy at Birth | 80.78 years | 72.30 years | 7.48 years higher in Sweden | 11.73% higher in Sweden |
| Crude Death Rate | 10.55 per 1,000 PY | 7.42 per 1,000 PY | 3.13 per 1,000 PY higher in Sweden | 42.18% higher in Sweden |
| Age-Standardized Death Rate | 7.37 per 1,000 PY | 11.88 per 1,000 PY | 4.51 per 1,000 PY lower in Sweden | 37.96% lower in Sweden |

**all indicators were estimated based on the entire age range.*

19. **If only 40% have no complications at the initial point of the analysis, and the "LE was 26.26 years (24.47-28.06) among males from the least deprived quintile, and 18.72 years (17.50-19.95) among males from the most deprived quintile." it is rather unexpected to see that "males from the least deprived quintile were expected to spent 9.31 years (7.50-11.12) without complications while males from the most deprived quintile were expected to spent 4.85 years (3.64-6.07) without complications". Please check the correctness of calculations for the years spent without complications.**

We assume that the editor’s statement “only 40% have no complications at the initial point of analysis” refers to Table 1. This conclusion is not correct as Table 1 indicates that this value corresponds to the entire study population (median age: 59.6 years, IQR: 54.3 - 66.9). As indicated in S4 Table, the corresponding prevalence of no complications at ages 45-54 (typically referred to as “weights” in the literature) was around 60% for all males and females. In a markovian multistate model, the time spent in one particular state is not per se defined by the prevalence of this particular state at a particular age or point in time. Instead, the time spent in this state is defined by the transitions occurring out of this state into other states (van den Hout et al. 2014).

In contrast to a parametric two-state survival model or the classic life table approach, the level of total LE in a markovian multistate model is not defined by mortality alone. Instead, it is defined as the sum of years spent in all transient states (van den Hout et al. 2014).

In our paper, we followed the best practice approach discussed in the literature for evaluating the correctness of calculations for a multistate survival model by comparing our estimates for total life expectancy with different approaches to estimate life expectancy (Chan et al. 2019; van den Hout et al. 2014). The results of these tests (S2 Fig, S3 Fig) validate our results. In other words: if one of our estimates for the number of years spent in a particular state would be completely off, this would become evident in either S2 Figure or S3 Figure by a strong deviation from the other approaches available for estimating LE.

We examined the mathematical correctness of our calculations in the statistical code and can confirm that our results are correct. Small deviations from results obtained via the parametric two-state model and the classic life-table approach are common and expected (Chan et al. 2019)^[5]^. This is the most appropriate validation for correctness of estimates and we now state that we follow the best practice approach described in the literature for validating the correctness of life expectancy estimates obtained from multistate survival models.

20. **The discussion has to be substantially reviewed, including: - appropriate placement of the "Limitations" section; - appropriate consideration of literature indicated in the "Introduction" and other available world data; - provision of hypotheses why diabetic complications in type 1 DM vary so prominently between social deprivation quintiles, while the Scotland has universal health coverage that should reduce 4-fold difference in mortality rates. If the HRs of transitions changed only marginally when controlling for differences in the distribution of risk factors, which exactly mechanisms could be responsible for the observed differences between SIMD quintiles? I.e. if HbA1c concentration introduced in the model does not influence too much the outcome in persons with diabetes and thus supposed to be comparable between SIMD quintiles, which factors could be responsible for the differences? Moreover, this finding makes necessary to provide in a main text a table with all clinical and biochemical parameters analysed in this manuscript for each SIMD quintile;- possible discussion of the literature considering the international studies aiming to reduce social inequalities for improving outcomes of DM type 2 and other (not DM) diseases.**

Thank you very much for this comment. We have now provided a set of comprehensive tables including all health information utilized for the sensitivity analysis by SIMD quintile (S7a – S7e Tables). As the focus of the paper is on population-level metrics rather than socioeconomic differences across various clinical parameters, we chose to place the respective tables in the supplementary material. We refer to these tables briefly in the section presenting results of the respective sensitivity analysis.

We have revised all sections of our discussion carefully and ensured that the covered content follows the STROBE Checklist. We now discuss our results more explicitly considering the literature mentioned in the introduction. At multiple points in our discussion, we now provide a stronger linkage of our results to mortality patterns observed in the general Scottish population. This gives an important piece of context to our results and strengthens the demographic focus of our paper. For example, it is remarkable but clearly not surprising that the SIMD LE gradient in the Scottish population with type 1 diabetes is nearly the same as the gradient observed among the general Scottish population.

We have now moved the strengths and limitations section to the end of the discussion.

This response corresponds to comment #5 provided by the expert reviewer.

21. **The figures are nice, but more figures should be introduced to represent not only the frequency of the number of complications across ages and sex groups, but also the prevalence of each complication (cardiovascular, diabetic foot, kidney etc.).**

Thank you for this suggestion. We now provide an additional figure in the supplementary material (S1 Fig) which details the prevalence of each complication over age for males and females of all SIMD quintiles.

22. **Please carefully revise all references, some of them have no year of publication (for example, ref 21)**

We have added the year for this publication and added missing/additional information for a small number of other references.

**Reviewers' comments:**

**Hohn et al. conducted a retrospective cohort study that aimed to estimate life expectancy (LE) for the Scottish population with type 1 diabetes at age 50 and to examine how many years of life were spent with and without the most common complications of diabetes. Investigators used data from about 8000 individuals with T1D from the Scottish Care Information-Diabetes database, a nationwide register for diabetes. Main exposures associated with LE were the number of complications and the social gradient, assessed using the Scottish Index of Multiple Deprivation. As expected, investigators reported significant gaps in LE between individuals developing the most complications vs individuals developing few complications. Gaps in LE were also observed between most and least deprived areas.**

**This is an interesting study that provides further evidence on the impact of the social gradient on health, with a particular scope on individuals with T1D in Scotland.**

**Methods used in the papers appear to be state of the art approach. I particularly appreciated the quality of the transition-specific models.**

Thank you very much for this well-balanced summary and the positive feedback. Your comments have prompted us to make further improvements to the description of our data, the methodology, and the validation of the obtained multistate model LE estimates.

**However, a few elements need clarifications:**

1. **The SCID was implemented in 2004. However, January 1st 2013 was used as the study baseline. Investigators are invited to clarify why they did not include all individuals 50+y of age from 2004 instead of limiting the sample to 2013+. The sample size, with n=about 8000 appears to have been sufficient for the analyses, but it is likely that many more individuals could have been included. Please clarify.**

The reviewer is completely right that the utilized data would have allowed a longer study period and that this would have resulted in a larger sample size. However, we explicitly limited our study baseline to 2013 because we wanted the analysis to reflect the most recent period. There have been important shifts in risk factor management and LE in diabetes in the past few decades and we are primarily interested in the contemporaneous situation.

Furthermore, covering a shorter period enabled a better comparison with the corresponding general population because National Records of Scotland report national LE/ HLE estimates for Scotland using a 3-year period. We have provided a clear justification for the choice of study period in the revised the introduction. This comment has prompted us to consider a follow-up study examining changes in life expectancy among males and females with type 1 diabetes in Scotland over time by using a contour decomposition approach examining the contribution of particular age groups and causes of death (Jdanov et al. 2017).

2. **How did authors manage potential change in SIMD for the same individual?**

Please see comment #5 from the academic editor.

3. **Authors suggest that they report a socioeconomic gap in the proportion of individuals without complications (Figure 1). While this is true for the 2 examples they mention in the text, mainly males/females aged 50-54, this difference is less evident as age increases. In females with 0 complication, from 64y+, there is no longer a marked socioeconomic gap. It if felt that the readers would benefit from this specification. Also, authors are invited to discuss the age X SIMD interaction in complication burden.**

Thank you very much for this comment and we fully agree. In the results section “Number of complications”, we now discuss Figure 2 (former Figure 1) in more detail. We have mentioned the observed inconsistent patterns with respect to the prevalence of females without complications at older ages. We have discussed the age X SIMD interaction regarding 1, 2, and 3+ complications. While there was no clear SIMD gradient over age with respect to 1 complication, there was a more consistent SIMD gradient over age with respect to 2 and 3+ complications.

4. **Figure 2 and elsewhere: authors are invited to present the number of individuals included in each of the 5 SIMD levels. Authors mentioned that the used SIMD quintiles: are they referring to quintiles within their study sample or quintiles within the SIMD index? While there are marked gaps between level 1 and level 5 exemplified in figure 2, it is unclear whether this reflect a significant proportion of the population or only extreme examples of poverty and wealth. I stress that I do not want to decrease the focus on the most deprived areas. However, such data would help to contextualize what are the socioeconomic gap authors are referring to.**

Thank you very much for this very interesting comment. We now report the size of each quintile in the main manuscript, Table 1. In addition, we have revised the first paragraph in the Section “Definition of covariates and complications” to provide more information on the SIMD 2016.

We used the original SIMD 2016 index and did not create the quintiles ourselves. Based on total population data and capturing the entire age range for a number of domains, the SIMD 2016 ranked 6,976 data zones of about 700 individuals each. Based on this ranking, 5 quintiles were created by the Scottish Government with every quintile reflecting *exactly* 20% of all data zones. This corresponds to one SIMD quintile covering *approximately* 20% of all individuals alive and residing in Scotland. However, as we focus on the population with type 1 diabetes in Scotland aged 50+, small differences in the size of quintiles can be expected. However, the SIMD was designed and intended to be used to reflect deprivation at the national level which would be distorted if we had created quintiles based on our study population.

5. **Authors mention that the LE gaps they reported among individuals with T1D is similar to LE gaps in the general Scottish population. It would be interesting to develop more on this similarity. One would have expected that the LE gaps associated with T1D would have been more important than the general population.**

We fully agree with the reviewer and would like to draw your attention in addition to comment #20 provided by the academic editor. At multiple points in our discussion, we now provide a much stronger link to mortality patterns observed in the general Scottish population pointing out the demographic focus of our paper.

**Journal Requirements:**

**When submitting your revision, we need you to address these additional requirements.**

1. **Please ensure that your manuscript meets PLOS ONE's style requirements, including those for file naming. The PLOS ONE style templates can be found at** [**https://journals.plos.org/plosone/s/file?id=wjVg/PLOSOne_formatting_sample_main_body.pdf**](https://journals.plos.org/plosone/s/file?id=wjVg/PLOSOne_formatting_sample_main_body.pdf) **and** [**https://journals.plos.org/plosone/s/file?id=ba62/PLOSOne_formatting_sample_title_authors_affiliations.pdf**](https://journals.plos.org/plosone/s/file?id=ba62/PLOSOne_formatting_sample_title_authors_affiliations.pdf)

We have revised all relevant aspects of our manuscript to comply with PLOS ONE’s style requirements to the best of our knowledge.

2. **Thank you for stating the following in the Competing Interests section: All authors have completed and submitted the ICMJE Form for Disclosure of Potential Conflicts of Interest. TMC reports grants from Diabetes UK Grant: 18/0005786, during the conduct of the study. SHW reports meeting attendance supported by Novo Nordisk, outside the submitted work. RJM reports personal fees from Sanofi, personal fees from NovoNordisk, outside the submitted work. NS reports personal fees from Amgen, personal fees from AstraZeneca, grants and personal fees from Boehringer Ingelheim, personal fees from Eli Lilly, personal fees from Novo Nordisk, personal fees from Pfizer, personal fees from Sanofi, outside the submitted work; HMC reports grants, personal fees and other from Eli Lilly and Company, during the conduct of the study; grants from AstraZeneca LP, other from Novartis Pharmaceuticals, grants from Regeneron, grants from Pfizer Inc., other from Roche Pharmaceuticals, other from Sanofi Aventis, grants and personal fees from Novo Nordisk, outside the submitted work. No other disclosures were reported.	Please confirm that this does not alter your adherence to all PLOS ONE policies on sharing data and materials, by including the following statement: "This does not alter our adherence to PLOS ONE policies on sharing data and materials.” (As detailed online in our guide for authors http://journals.plos.org/plosone/s/competing-interests). If there are restrictions on sharing of data and/or materials, please state these. Please note that we cannot proceed with consideration of your article until this information has been declared.	Please include your updated Competing Interests statement in your cover letter; we will change the online submission form on your behalf.**

We have provided a revised competing interests statement in our cover letter.

3. **We noted in your submission details that a portion of your manuscript may have been presented or published elsewhere. [We confirm that this manuscript and its content is neither submitted/under review nor has been published elsewhere. An abstract with preliminary findings has been accepted for an oral presentation at the EASD 2021 Annual Meeting (28.09.2021 – 01.10.2021)] Please clarify whether this [conference proceeding or publication] was peer-reviewed and formally published. If this work was previously peer-reviewed and published, in the cover letter please provide the reason that this work does not constitute dual publication and should be included in the current manuscript.**
   We have provided a revised statement on dual publication in our cover letter and the online submission system.
4. **In your Data Availability statement, you have not specified where the minimal data set underlying the results described in your manuscript can be found. PLOS defines a study's minimal data set as the underlying data used to reach the conclusions drawn in the manuscript and any additional data required to replicate the reported study findings in their entirety. All PLOS journals require that the minimal data set be made fully available. For more information about our data policy, please see** [**http://journals.plos.org/plosone/s/data-availability**](http://journals.plos.org/plosone/s/data-availability)**.	Upon re-submitting your revised manuscript, please upload your study’s minimal underlying data set as either Supporting Information files or to a stable, public repository and include the relevant URLs, DOIs, or accession numbers within your revised cover letter. For a list of acceptable repositories, please see http://journals.plos.org/plosone/s/data-availability#loc-recommended-repositories. Any potentially identifying patient information must be fully anonymized.	Important: If there are ethical or legal restrictions to sharing your data publicly, please explain these restrictions in detail. Please see our guidelines for more information on what we consider unacceptable restrictions to publicly sharing data: http://journals.plos.org/plosone/s/data-availability#loc-unacceptable-data-access-restrictions. Note that it is not acceptable for the authors to be the sole named individuals responsible for ensuring data access.	We will update your Data Availability statement to reflect the information you provide in your cover letter.**

We have updated our data availability statement in the online submission form in line with the guidelines detailed in both links. We also provide the revised data availability statement in our cover letter.

5. **One of the noted authors is a group or consortium [SDRN-Epi]. In addition to naming the author group, please list the individual authors and affiliations within this group in the acknowledgments section of your manuscript. Please also indicate clearly a lead author for this group along with a contact email address.**

We have revised the title page and acknowledgements of our manuscript. We now state clearly that SDRN Epi is a group/consortium. We now also provide information on the authors and affiliations within this group and indicate Helen m Colhoun as the lead author for which we provide an email address.

**References:**

Chan MS et al. (2019) Socio-economic inequalities in life expectancy of older adults with and without multimorbidity: a record linkage study of 1.1 million people in England International journal of epidemiology 48:1340-1351

Colombo M et al. (2020) Comparison of serum and urinary biomarker panels with albumin/creatinine ratio in the prediction of renal function decline in type 1 diabetes Diabetologia 63:788-798

Datta S, Satten GA (2001) Validity of the Aalen–Johansen estimators of stage occupation probabilities and Nelson–Aalen estimators of integrated transition hazards for non-Markov models Statistics & probability letters 55:403-411

Jdanov DA, Shkolnikov VM, van Raalte AA, Andreev EM (2017) Decomposing current mortality differences into initial differences and differences in trends: the contour decomposition method Demography 54:1579-1602

Krolewski AS (2015) Progressive renal decline: the new paradigm of diabetic nephropathy in type 1 diabetes Diabetes care 38:954-962

Levey AS et al. (2009) A new equation to estimate glomerular filtration rate Annals of internal medicine 150:604-612

Preston SH, Heuveline P, Guillot M (2001) Demography : measuring and modeling population processes. Blackwell Publishers, Malden, MA

van den Hout A, Chan MS, Matthews F (2019) Estimation of life expectancies using continuous-time multi-state models Computer methods and programs in biomedicine 178:11-18

van den Hout A, Ogurtsova E, Gampe J, Matthews FE (2014) Investigating healthy life expectancy using a multi-state model in the presence of missing data and misclassification Demographic Research 30:1219-1244

Vandenbroucke JP et al. (2007) Strengthening the Reporting of Observational Studies in Epidemiology (STROBE): explanation and elaboration PLoS medicine 4:e297

1. <https://journals.plos.org/plosmedicine/article/file?id=10.1371/journal.pmed.0040297&type=printable> Accessed: 08/12/2021
2. <https://www.isdscotland.org/Products-and-Services/GPD-Support/Deprivation/SIMD/_docs/PHI-Deprivation-Guidance.pdf> (see: page 10/11). Accessed: 08/12/2021
3. Scottish Diabetes Data Group (2017) Scottish Diabetes Survey 2017. Available from: <https://www.diabetesinscotland.org.uk/wp-content/uploads/2020/02/Diabetes-in-Scotland-website-Scottish-Diabetes-Survey-2017.pdf> Accessed: 08/12/2021
4. Sweden:	<https://data.worldbank.org/indicator/SP.DYN.LE00.FE.IN?locations=SE>

   Kazakhstan:	<https://data.worldbank.org/indicator/SP.DYN.LE00.FE.IN?locations=KZ>

   both aaccessed: 08/12/2021
5. See: Supplementary Data, Appendix E, Table E3
