# Supplementary material for: Large socioeconomic gap in period life expectancy and life years spent with complications of diabetes in the Scottish population with type 1 diabetes, 2013–2018
Source: PLoS One. 2022 Aug 11;17(8):e0271110. doi: 10.1371/journal.pone.0271110 (PMC9371295; doi:10.1371/journal.pone.0271110)
Supplement: Attachment — Submitted filename: 2 Respose to Reviewers.docx [file pone.0271110.s015.docx]

**Response to Reviewers**

We would like to thank both expert reviewer and the academic editor for the positive, constructive, and helpful feedback. All changes we have made are summarized below. In case we were not able to fully incorporate a comment, we have provided a clear justification for this.

**Comments from the Academic Editor**

**Congratulations for the analysis and all work you have performed on the manuscript. The changes you have introduced, and the provided explanations are very welcome and substantially improved the manuscript.**

**However, several issues remained and need to be addressed in the manuscript**

**1. Regarding the rationale provided in the answers to the comment 8 (criteria for the CKD detection), there are following considerations:**

**- It remains unclear whether the 2 confirmations of** **initial abnormally low eGFR have been considered to correctly classify a patient, as required by the KDIGO guidelines.**

The data cleaning algorithm which is currently in place for eGFR identifies single acute transient drops in eGFR and excludes them. These single acute transient drops are therefore not considered when defining CKD. We did not apply any further criteria, such as 2 conformations of initially initial abnormally low eGFR. However, we have now confirmed that of all those defined as having baseline prevalent CKD or incident CKD during follow up, 83% had more than one GFR<60ml/min/1.73m^2^ at least 3 months apart with a further 10% having had a second record <75 /min/1.73m^2^ and have stated this in the results section.

**- You referred to Krolewski 2015 to rationale that ACR lacks specificity and sensitivity for progressive decline in eGFR. Of note, the Krolewski's paper refers to DM type 1 while obviously the DM type 2 is predominant in the studied population.**

We would like to emphasise that, as indicated by the title of this study and throughout the manuscript, we only included individuals with a confirmed diagnosis of type 1 diabetes in our study.

**Moreover, the huge amount of other literature exists (including current guidelines) indicating that ACR is crucial in diabetic nephropathy evaluation. Please reflect these sources for providing equilibrated view. You referred to your own data indicating "The majority (59%) of those with chronic kidney disease stages G3–G5 did not have albuminuria on the day of recruitment or previously (Colombo et al. 2020)". Hopefully that means the ACEIs/ARBs work! (in constellation with genetic and other factors) GFR decline (especially 3a) could be related to age changes, and we also do not expect that a patient with diabetes should absolutely has diabetic nephropathy with elevated ACR. Moreover, non-diabetic nephropathies are prevalent in this population.**

We fully agree with the editor that ACR is a hugely important marker of and risk factor for renal function decline. However, even those without albuminuria can progress to renal failure so we do not require ACR to be present to define CKD as is consistent with KDIQO (de Boer et al. 2020). To make clear that we fully acknowledge the importance of albuminuria and to acknowledge how our definition relates to KDIQO nomenclature we have now inserted the following sentence into the methods section:

*“The Kidney Disease: Improving Global Outcomes (KDIQO) Clinical Practice Guideline for Diabetes Management in Chronic Kidney Disease defines CKD as abnormalities of kidney structure or function, present for >3 months, with implications for health [27]. Given the importance of albuminuria in predicting a more rapid renal function decline at any eGFR level CKD is further classified based on Cause, GFR category (G1–G5), and Albuminuria category (A1–A3). Here for simplicity, we define CKD as ever had a record of an estimated glomerular filtration rate (eGFR) of < 60 mL/min/1.73 m^2 or being in receipt of renal replacement therapy (i.e., KDIQO G3a or worse). We did not require the presence of albuminuria, but we provide data on the coexistence of albuminuria in the results section. Transient acute falls in eGFR were not included as CKD. All values for eGFR are based on the equation provided by the Chronic Kidney Disease Epidemiology Collaboration (CKD-EPI) using serum creatinine [27].”*

Then in the results section we state: *“We note that of those defined as ever having had CKD by end of follow up, 83% had more than one eGFR <60 at least 3 months apart thus meeting the KDIQO definition with a further 10% having had a second record <75 /min/1.73m2 and 39 % had a history of moderately (3–30 mg/mmol) and 21% a history of severely (>30 mg/mmol) increased albuminuria.”*

Furthermore, in the limitations section we now state that: *“We fully acknowledge that all four complications could have defined differently. Considering this aspect, we acknowledge that our definition of CKD is less stringent than the KDOQI definition.”*

**Finally, these considerations should be substantially revised. I didn't pass through the whole manuscript to see how you reflected these ideas, and in general it would be easier to estimate the changes you have performed if you will cite them directly in Q&A sections, instead of highlighting the changed text over the 30 pages of the manuscript.**

We fully agree and have now included direct citations in all sections to simplify the review process.

**2. Please indicate how you managed the analysis in the case of a person changed the residence from more prominent to less prominent social deprivation area defined by SIMD. You explained that the 2016 release was used consistently throughout the entire study period that is perfectly fine, but it remained unclear how was accounted a person who lived in place A with low SIMD and during the study transferred to place B with higher SIMD.**

Using the 2016 SIMD release consistently throughout the study means that we used this information as a time-constant variable for all individuals. The reason for this is that each SIMD release presents a one-off, cross-sectional piece of information. Based on this cross-sectional character, it is therefore not possible to observe a change in SIMD quintile. Changes with respect to the SIMD quintile of individuals will only ever be visible if at least two releases are used. In our study, we utilize the SIMD appropriately and as suggested by the Information Services Division (ISD) of NHS Scotland ^[1]^. We incorporated the following statement into the revised manuscript on page 8: *“In line with recommendations by ISD for the correct use of the SIMD, we used the SIMD 2016 release consistently throughout the study period; and as a time-constant variable. As we used one SIMD release consistently throughout the study period, there were no changes in SIMD quintile for the studied individuals.”*

**3. You provided very useful details for the states transition but named the states just numerically. It would be much easier to provide some meaningful names instead of State 1 – State 3, etc.**

We assume that this comment refers to the section “Multistate Approach” on page 9. We now state explicitly what these states mean: *“Depending on the number of complications at time of study entry, individuals started either in the state of ‘no complication’ (state 1), ‘1 complication’ (state 2), ‘2 complications’ (state 3), or ‘3+ complications’ (state 4). (…) This means that we did not allow the following transitions: ‘no complication’ to ‘2 complications’ (from state 1 to state 3), ‘no complications’ to ‘3+ complications’ (from state 1 to state 4), and from ‘1 complication’ to ‘3+ complications’ (state 2 to state 4).”*

**4. Regarding the individual/population level considered in the comment 13 - I agree with your arguments, and completely understand that development of the individual-level decision making tool will require much more efforts and control. However, the population-level metrics are based on the individual-level estimates. You have proposed a very nice and interesting approach, but because of its complexity the exact calculations are not very clear. Due to this, please provide examples of 2-3 individual-level calculations - thus the readers could have more insights into the methodology.**

In our statistical approach, we do not derive individual-level estimates. All estimates reflect a population-level perspective. To clarify this, we have now provided a very detailed and step-by-step example documenting how we derived our population-level metric, including all related calculations and the corresponding R-Code (S1 Text in the Supplementary Material – see also comment 4). The illustrated example details the exact procedure to derive the estimates shown in Figure 4. This example ensures that interested readers can replicate and utilize our methodology. We have included the following statement in our main manuscript on page 12: “*A detailed example documenting our calculations is presented in S-1 Text.”* Unfortunately, and due to data protection regulations, we were not able to share the underlying data or data objects.

**5. Regarding data representation (Me and IQR vs X (SD)) - please leave normally distributed variables (like age) as X (SD). My comment for the Me and IQR concerns only the description of diabetes duration and other variables with not normal distribution. Regarding follow-up time over the entire 6-year period, please check the correctness of the calculations. The median 6.0 (IQR of 6.0 6-0) years would mean very low mortality, and inclusion of the majority of patients since from the beginning of the follow-up. Usually, patients with diabetes are of high risk, and thus have elevated mortality, and more information of the reasons for the end of follow-up for 1822 (almost 20% of the studied population) should be provided, probably even better to present the mortality rate.**

Based on statistical tests, we found that all relevant variables - age, diabetes duration, and follow-up time - are not normally distributed. As requested, we therefore provided the median and the interquartile range for the variables which are not normally distributed.

We have revised the title of Table 1 on page 13 and included the following statement: *“Out of all 1,822 individuals which were not observed for the entire 6-year period, 1,495 individuals died during the follow up, and 327 individuals were censored as they became unobservable for other caused than death, such as out-migration.”*

We present detailed mortality rates by sex, SIMD quintile, and 5-year age groups in Figure 3. We can reassure the editor of the correctness of our mortality estimates. Our mortality estimates presented in Figure 3 are very close to estimates we previously reported for the Scottish population with type 1 diabetes, reflecting the period 2008-2010 (see: Livingstone et al. 2010; Table 1, 5-year age groups starting from Age 50-54 onwards). We would like to point out to the editor that a median follow-up time of 6y with an IQR of 6.0y – 6.0y can technically still be achieved by study population which is subject to a 24.99% probability of dying within this period. Please bear in mind that this would be an unrealistically high probability of dying for a population with type 1 diabetes - given the mean age of 60 years. One of the major reasons behind the consistently long time of follow-up for most individuals is that we used routinely collected, administrative healthcare records for our study. This substantially reduced panel attrition etc. which is commonly observed in surveys.

**6. Regarding the life expectancy and mortality considered in the comment 18, it is very nice to see the literature-based example, but the raised issue was related to the exact findings in the manuscript. Moreover, in the answer you have referred to both "Remaining life expectancy was calculated at age 50" and "life expectancy at birth" that somewhat confusing because only one indicator is considered in the manuscript. Finally, to resolve any doubts, please provide in the manuscript the table with the calculations you made based on the literature data (i.e., mortality rates, life expectancy), but using instead of "Sweden" and "Kazakhstan" column data from the analysis for "males" and "females".**

We provided this table in our last response to illustrate that absolute and relative differences in demographic measures of mortality are not comparable.^[2]^ For example, the mortality rate at age x is only dependant on the population at risk and the number of deaths at age x. In contrast, life expectancy at age x is dependent on mortality patterns observed at age x, x+1, x+2, x+3, … up to the highest age group. While we performed all relevant calculations (see table below), we decided to not include this table in the supplementary material or main manuscript. In our opinion, such a comparison is (A) not required given the scope of the paper, and (B) would require detailed formal-demographic context which is beyond the scope of this paper.

For our main results, we now provided a detailed example of our calculations in S1 Text in the Supplementary Material – see also comment 4. We carefully examined our manuscript and can guarantee that we always refer to LE at age 50 – see also comment 8.

| Sex | SIMD | e(50) | m(50-54) | CDR(50+) | ASCDR_EU(50+) |
| --- | --- | --- | --- | --- | --- |
|  | Quintile |  | per 1,000 PY | per 1,000 PY | per 1,000 PY |
| Females | All | 25.10 | 21.47 | 31.22 | 38.13 |
| Males | All | 22.18 | 22.01 | 34.25 | 48.33 |
| Females | Q1 | 21.17 | 29.86 | 41.55 | 51.65 |
| Females | Q2 | 23.70 | 19.82 | 35.47 | 45.91 |
| Females | Q3 | 25.46 | 24.98 | 31.31 | 36.36 |
| Females | Q4 | 26.94 | 13.59 | 27.69 | 32.35 |
| Females | Q5 | 29.32 | 19.89 | 21.60 | 28.23 |
| Males | Q1 | 18.72 | 39.19 | 44.26 | 67.14 |
| Males | Q2 | 21.19 | 22.37 | 38.31 | 53.13 |
| Males | Q3 | 22.32 | 23.89 | 34.25 | 45.11 |
| Males | Q4 | 23.57 | 15.43 | 31.42 | 46.77 |
| Males | Q5 | 26.26 | 9.48 | 24.50 | 34.41 |
| With:  e(50): life expectancy at age 50 | | |  |  |  |
| m(50-54): mortality rate at ages 50-54 | | | |  |  |
| CDR(50+): crude death rate for the ages 50+ | | | | |  |
| ASCDR_EU(50+): CDR for the ages 50+ based on European Standard Population 2018 Revision^[3]^ | | | | | |

**7. You provided very useful explanations in the comment 19, and there are no doubts about the robustness of the calculations. But please provide examples of 2-3 individual-level calculations - thus the readers could have more insights into the methodology of the multistate survival model. It seems that having the ready statistical code and all the data it will not take much time but will demonstrate the readers how the exact numbers were calculated.**

We assume that this comment of the editor refers to the presented total and state-specific estimates in Figure 4 and Figure 5. Unfortunately, we cannot share the exact data or underlying data objects due to data protection regulations. Furthermore, we did not derive individual-level estimates and all estimates presented in this paper reflect the population level.

We now provide a detailed example of our calculations with the corresponding R-Code (S1 Text in the Supplementary Material – see also comment 4). We have included the following statement in our main manuscript on page 12: “*A detailed example documenting our calculations is presented in S-1 Text.”*

**8. I could not find the figures titles in the main manuscript. Please check whether they are present in the file. Please also define clearly in the figures captions that LE refers to LE at 50 (both in the main manuscript and the supplementary material).**

Figure titles are provided in the main manuscript within the main text. We now also included an overview of all captions of tables and figures. All relevant captions indicate that our results reflect LE at age 50:

*“Fig 4: Total and state-specific life expectancies at age 50 among males and females of the Scottish population with type 1 diabetes.”*

*“Fig 5: Total and state-specific life expectancies at age 50 among males and females of the Scottish population with type 1 diabetes by SIMD quintile.”*

*“S2 Fig: Comparison f estimates for Life Expectancy at age 50 for males and females obtained from the multistate survival model (main findings presented in the paper) with corresponding LE estimates we derived using the Chiang (1984) life table method and a parametric two-state survival models.”*

*“S3 Figure: Comparison f estimates for Life Expectancy at age 50 for males and females by SIMD quintile obtained from the multistate survival model (presented in the paper) with corresponding LE estimates we derived using the Chiang (1984) life table method and parametric two-state survival models.”*

**9. You have produced very informative figures and tables for the Supplementary materials. However, each of them presented in a separate file that makes their downloading and revision extremely uncomfortable. Please prepare a single file with all Supplementary materials for the revision, you could use a free tool like pdfSam to merge all of them.**

Thank you very much for pointing this out. We have combined all supplementary material in one file.

**Finally, I would like to congratulate you with the work you have performed for the sophisticated analysis of the very interesting data. Improving the several remaining points indicated above would be of great value for the future readers of the manuscript. Receiving from you the revised version, it would be possible to evaluate through the whole manuscript.**

We would like to thank the academic editor for his positive and constructive feedback on our revised manuscript. We hope that all comments were addressed satisfactorily. We hope that in particular our example calculations have convinced the editor of the quality and correctness of our results.

**Reviewer #1 comments**

**I reviewed the revised version of the manuscript as well as the responses to the comments. All my questions have been adequately addressed. I have no further comments.**

Thank you very much for your comments and positive feedback on our manuscript.

**Reviewer #2 comments**

**Thank you for the thorough response and changes. The revised manuscript is much stronger in the presentation of the methods and interpretation.**

We would like to thank Reviewer #2 for the positive feedback on our revised manuscript.

**Journal Requirements:**

**Please review your reference list to ensure that it is complete and correct. If you have cited papers that have been retracted, please include the rationale for doing so in the manuscript text or remove these references and replace them with relevant current references. Any changes to the reference list should be mentioned in the rebuttal letter that accompanies your revised manuscript. If you need to cite a retracted article, indicate the article’s retracted status in the References list and include a citation and full reference for the retraction notice.**

We have checked all cited references and can ensure that all material is cited correctly, can be easily identified, and hasn’t been retracted. All online resources are provided with a link as well as a date and time when we have accessed them last. Please let us know if this comment applies to any specific material we have cited in our manuscript.

**References**

de Boer, I. H., Caramori, M. L., Chan, J. C., Heerspink, H. J., Hurst, C., Khunti, K., ... & Rossing, P. (2020). KDIGO 2020 clinical practice guideline for diabetes management in chronic kidney disease. *Kidney international*, 98(4), S1-S115.

Livingstone, S. J., Levin, D., Looker, H. C., Lindsay, R. S., Wild, S. H., Joss, N., ... & Scottish Renal Registry. (2015). Estimated life expectancy in a Scottish cohort with type 1 diabetes, 2008-2010. *Jama*, *313*(1), 37-44.

Preston SH, Heuveline P, Guillot M (2001) Demography : measuring and modeling population processes. Blackwell Publishers, Malden, MA.

1. <https://www.isdscotland.org/Products-and-Services/GPD-Support/Deprivation/SIMD/_docs/PHI-Deprivation-Guidance.pdf>

   Accessed: 04/05/2022
2. A minor exemption applies only in stationary life table populations in which e(0) = 1/CDR (Preston et al. 2001) – a setting which is not given here due to (a) our cohort study design, and (b) the multistate framework
3. <https://www.opendata.nhs.scot/dataset/4dd86111-7326-48c4-8763-8cc4aa190c3e/resource/edee9731-daf7-4e0d-b525-e4c1469b8f69/download/european_standard_population.csv>

   Acessed: 04/05/2022
