# Supplementary material for: Large socioeconomic gap in period life expectancy and life years spent with complications of diabetes in the Scottish population with type 1 diabetes, 2013–2018
Source: PLoS One. 2022 Aug 11;17(8):e0271110. doi: 10.1371/journal.pone.0271110 (PMC9371295; doi:10.1371/journal.pone.0271110)
Supplement: Attachment — Submitted filename: 2 Respose to Reviewers.docx [file pone.0271110.s016.docx]

**Response to Reviewers**

We would like to thank the academic editor for the positive, constructive, and helpful feedback. The requested revisions were minor, all changes we have made are summarized below.

**Comments from the Academic Editor**

**Congratulations for the very interesting analysis and all work you have performed on the manuscript. The changes you have introduced, the additional results are informative and made the manuscript better. Sorry for the delay in replying to your revised version. There are only several minor items that should be corrected because the PLOS ONE production team informed, they require the very final version of the manuscript to proceed further. Please find below the list of minor items to be changes.**

**1. Please express numeric values of years of life and life expectancy with a complication with one decimal sign, since two decimal signs for years are excessive. For the same reason, I would suggest you remove the "years" word from the parenthesis with the 95%CI (like "29.32 years (95% CI: 27.51-31.13 years)" and elsewhere - the second "years" seems excessive)**

We have addressed this consistently throughout the manuscript and in all relevant figures. For all state estimates we now consistently present the number of years using one digit, including Figure 4 and Figure 5. The second phrase “year”, when providing 95% confidence intervals, has been dropped consistently throughout the manuscript.

**2. Line 61: please put the abbreviation KDIGO first, then the explanation.**

We have now introduced this abbreviation correctly in the overview of all utilized abbreviations (Line 61). We now specify this as:

“KDIQO - Kidney Disease: Improving Global Outcomes”

**3. In the Supplementary, please drop the "Comprehensive" word from the tables' titles.**

We have removed the word “comprehensive” from the titles of all S7 and S8 Tables.

**4. In the STROBE statement I would suggest you correct the referring from the pages number to the manuscript sections or limit the statement just with the "Yes" with possible details where necessary. Please note the final pdf version of the manuscript will have completely different page allocation, and the current version of the STROBE statement will not correspond to the pdf pages numeration. Another item concerning the STROBE Statement is the "Funding" details specified as "See online system", please be more specific.**

We have now included “Yes” rather than the exact page number. As requested, we have now also included detailed information on the funding. We now state: *“This study was supported by Diabetes UK grants: 17/0005627 - received by HMC and 8/0005786 - received by TMC. The funder had no role in designing the study or in analysing and interpreting data and results.”*

**5. Please change for the clarity the phrase introduced at lines 332-335, it is not clear why the threshold of 75 ml/min/1.73m2 has been selected in the part "with a further 10% having had a second record** **<75 /min/1.73m^2" - this value represents normal GFR in the absence of albuminuria. As a general idea apart of this manuscript, you have a nice data about the GFR and albuminuria that could be a topic of further manuscript based on the KDIGO-fully-compliant CKD definition, and another analysis investigating patients with a single abnormal GFR or albuminuria values.**

Thank you very much for pointing this out and thank you very much for your suggestions. To increase the readability of the sentence and as the information on further readings <75 /min/1.73m^2 are not of relevance in our context, we have now specified this sentence the following way:

*“We note that of those defined as ever having had CKD by end of follow up, 83% had more than one eGFR < 60 mL/min/1.73 m^2 at least 3 months apart, thus meeting the KDIQO definition, and 39% had a history of moderately (3–30 mg/mmol) while 21% had a history of severely (>30 mg/mmol) increased albuminuria.”*

**Again, congratulations to the performed analysis.**

Thank you very much for your constructive and positive feedback on our manuscript throughout the review process. We very much appreciate your positive decision.
